# The Effect of Lesions of the Oral Cavity upon Other Organs and the System at Large and the Relation of the Physician Thereto

**Published:** 1905-11

**Authors:** Price Cheaney

**Affiliations:** Dallas, Texas


### TEXAS MEDICAL JOURNAL.

ESTABLISHED JULY, 1885.

PUBLISHED MONTHLY.—SUBSCRIPTION $1.00 A YEAR.

VOL. XXI. AUSTIN, NOVEMBER, 1905. No. 5.

For Texas Medical Journal.

### The Effect of Lesions of the Oral Cavity Upon Other Organs and the System at Large and the Relation of the Physician Thereto.

BY PRICE CHEANEY, M. D., D. D. S., DALLAS, TEXAS.

In presenting this subject for your consideration this evening, I am persuaded that I am entering a field which has not had the attention from the medical profession at large that it should have had, and which will richly repay a close study from those to whom are confided the physical welfare of the public at large.

I can not, in the time which ought to be consumed by a paper of this kind, consider the subject exhaustively, but I desire to call attention to a few examples and trust that the experience of those present will help to develop the subject more fully.

When we consider the part which the mouth and teeth play in the economy of the human system and realize that the nourishment of the body is due to the proper digestion and assimilation of the nutritious elements contained in our food and that in order for the food to be properly digested it must be thoroughly triturated so as to expose every part of it to the action of the digestive fluids, and that the process of digestion itself commences in the mouth, through the action of the salivary fluids, we can see what an emphasis has been placed by nature herself on the organs of mastication, deglutition and insalivation.

The bad effects on the system of improperly masticated food, or of food mixed with destructive germs and pus, which are the result of uncleanly, diseased conditions of the oral cavity, is sometimes appalling, and it is gratifying to see the transformation which takes place, as a result of remedying diseased conditions of the oral cavity and restoring a normal, physiological condition of affairs. Cases of dyspepsia which have strenuously resisted a continued round of therapeutic agents, yield like dew before the sunshine, and every dental practitioner has seen cases of anemia, neurasthenia and even incipient tuberculosis, cured by simply purifying a mouth that was reeking with germs and placing the patient in a condition where he could properly prepare the food for his stomach.

In neurasthenia, probably one of the most puzzling conditions which the physician is called upon to treat, the influence of irritation from the oral cavity, caused by diseased teeth, alveolar abscesses, pyorrhea, etc., will frequently keep the patient in a state of nervous excitement, and undo the best directed efforts of the medical attendant; and a lack of ability to properly masticate the food will prevent his most skillful treatment from being crowned with success.

In speaking of the treatment of this disease, Hermann, that prince of writers on diseases of women, says: "There is no specific for neurasthenia. The treatment consists of removing the conditions which have exhausted the nervous energy, and placing the patient in circumstances which are favorable to his recovery." That this can not be done while there are continual causes for neuralgias and dental irritations, goes without saying, as one of the distinctive characteristics of this disease is the magnifying of every slight trouble and the inability to bear any kind of pain.

In the famous Weir-Mitchell treatment for this disease, great stress is placed upon the nourishment of the patient through natural means. In referring to the use of drugs for this purpose, Treves says, "You can not pour strength down the oesophagus." Obviously, then, the proper attention to the dental organs, so as to give the patient the opportunity to get the most good out of his proper nourishment, is of paramount importance, both as a prophylactic and a curative treatment.

In gastric catarrh, one of the most frequent causes is imperfectly masticated food. Einhorn says that "If food be imperfectly masticated and swallowed in big lumps, it may mechanically disturb the stomach, and lead to inflammation," and every physician is constantly called upon to treat catarrhal diarrheas caused by these unmasticated and consequently undigested lumps being passed into the intestines from the stomach.

In cases of gastric ulcer, the pain, caused by hard, unmasticated lumps coming in contact with the ulcerated parts, is sometimes excruciating.

On the other hand, Professor Garretson, in his great work on Oral Surgery, says, "Functional derangements of the stomach as the origin of reflex or radiated odontalgias are to be considered. Any one who has ever observed the relationship existing between the pneumogastric and third nerve, as manifesting stomachic derangement in the enlargement of the pupil of the eye, will be at no loss to associate the fifth and ninth nerves. Pure neuralgia, as the term has common significance, is, I am satisfied, a very rare affection; an aching nerve will be generally found to have a lesion outside of its so estimated idiopathic condition, and it can generally be found by looking closely for it." If this be so with regard to reflex odontalgias, may not the converse be frequently the case, and stomachic trouble be more frequently caused by lesions existing in the oral cavity than is generally supposed?

Ocular troubles due to radiation from the oral cavity through communicating branches of the fifth pair of nerves, are of common occurrence, and Professor Brubraker, in the *American System of Dentistry,* cites a case of amaurosis of twelve years' duration which was cured by extracting a tooth.

Gazelowski (*Revue generale d opthalmologie,* 1888), cites cases of rebellious corneal disease arising from reflex irritation induced by difficult eruption of teeth during first dentition which were cured by relieving the dental irritation. Dr. Geo. T. Stephens, of New York, in commenting upon it, however, takes issue with Gazelowski's conclusions, and explains it by saying that, "at the time of dentition, when unusual nervous energy is demanded, the entire supply is already in requisition for the ordinary purposes of the child, and in overcoming the difficulties of ocular adjustments, for these make great demands upon the child's nervous powers, hence the eruption of the teeth is difficult, not necessarily because of unusual resistance of tissue overlying them, but because of inadequate nervous force to push them out and heal the wound. The irritation of this arrested process is felt now, in addition to the occular irritation, and the reaction which sometimes occurs elsewhere, is located in the eye itself. Now, relief may be had by removing the primary exhausting or the resulting irritating cause. In either case, after the removal of the one, be it the primary or the secondary cause, there is a nervous reserve sufficient to heal the local disorder." No matter which of these learned gentlemen was correct in their deductions, the relationship between the oral cavity, the general system and the eye is clearly established.

Dr. Stephens also recites a case in which he had previously had a tooth removed for supposed abscess, when the cause was severe eye strain, and relates, in addition, cases where teeth had been removed when the real cause of pain was glaucoma. These cases of dental pain caused by the eyes are, however, more rare than eye trouble caused by reflex dental irritation.

Every physician is, of course, aware of the effect of dentition on the general system and the nervous disorders resulting therefrom.

Aural troubles are frequently the result of extension of irritation of the buccal mucous membranes, incident to the eruption of wisdom teeth, to the nasopharynx and the consequent involvement of the eustachian tube, or in the form of neuralgias through communicating branches of the fifth pair of nerves.

The tonsils, while not frequently affected by extension of irritation from the buccal membranes, may be the seat of trouble which has its starting point there, especially where there is a tendency to recurrences of tonsillar troubles.

The nasal cavity is frequently affected by direct extension through the palate, but more frequently as a result of trouble located in the antrum, which has its origin in alveolar abscesses occurring on devitalized teeth.

Antral trouble, however, I am convinced is much more rare than is generally supposed by the profession, and a great many cases of supposed abscess of the antrum are in reality cases of alveolar abscess, and frequently disappear upon proper treatment being directed to this trouble.

The conditions found in the mouth are frequently the first indications of systemic conditions. Of course, all are familiar with Hutchinson teeth as an indication of congenital syphilitic taint; with mucous patches in the mouth in syphilis; and with the condition of the gums and salivary glands in cases of ptyalism, but all are not so well acquainted with the fact that a tendency to pyorrhea alveolaris is sometimes the first visible evidence of an excess of uric acid in the system; and it is gratifying to see the benefit which occurs to the general health when uric acid is combatted in the treatment of cases of pyorrhea.

A point of great interest to physicians is the condition of the fluids of the mouth during pregnancy. It is a well known fact that teeth decay more rapidly during the pregnant condition than at other times. This has given rise to the old saying, "Every baby costs a tooth." Would to God, it were only one! This has been attributed to the supposed appropriation by the child of the lime salts contained in the teeth of the mother. With all due deference to authorities, I wish to take exception to this view, and cite the fact that in no part of a tooth is its microscopical structure analagous to bone, which is subject to being robbed of its limy structure, except in the cementum or crusta petrosa, covering the root of the tooth, and lime salts once taken into the dentine or enamel of a tooth are never taken away except by decay. This accounts for the fact that teeth continue to get darker from childhood to the grave from the continued deposition of lime salts. The real cause of the increased decay in the teeth of pregnant women, in my judgment, is that the chemical condition of the saliva is changed, being rendered either more acid or more alkaline, by the condition of the general system, and this, in turn, furnishes a pabulum for the micro-organisms of decay. From the fact that acids will dissolve bone substance, and the additional fact that some of our scientists have shown by their researches that some kinds of decay are caused by lactic acid which is the result of the ptomaines of oral bacteria, it would seem that an acid condition of the fluids of the mouth would favor decay, but my own observations for the past twenty years, has been that where there is extensive decay in the teeth of pregnant women, there is invariably an alkaline condition of the saliva, as shown by a litmus paper test, and on several occasions I have been able to make a diagnosis of pregnancy, from the general existence of the peculiar white decay, which we find in the mouths of pregnant women, accompanied by an extremely alkaline condition of the saliva, when the husbands of the ladies in question were in doubt as to whether the condition existed.

As a practical application of this theory, I have found that decay could be largely controlled by the free use of pickles, lemonades and other acids when extreme alkalinity existed, and by the use of soda bi carb. when there was undue acidity. It is during the time of pregnancy, however, that, unless driven to him by actual pain, the dental specialist sees very little of his lady patients, while the family physician is frequently consulted; hence the necessity of careful watch in this particular by the attending physician.

Dental irritations, and especially an alveolar abscess, may f[illegible]quently be the complicating "straw that breaks the camel's back"

and gives a fatal ending to what would otherwise be a happy recovery, but the absolute inability to undergo dental treatment while in an enfeebled condition caused by protracted illness, prevents the removal of this complication while in the hands of the physician.

Serious results frequently come from the neglect of seemingly trivial things in the mouth. The writer has known a neglected deciduous tooth to abscess, destroying the germ of the permanent tooth, and finally result in a case of osteo-myelitis, necessitating a serious operation on the inferior maxillary, endangering the life of the patient, when all could have been prevented by proper attention in time to the temporary tooth. He has been obliged to remove large sequestra of necrosed bone from the jaws, and has seen life scars result from neglected teeth which have abscessed. Neglected decay in teeth frequently causes abscesses, which, in turn, affect the cavity of the antrum, extending from there to the nose and throat and frequently affecting the eye. Numbers of persons go for months and years whose mouths are filled with accumulations of tarter, keeping the gums in a turgid, inflamed condition, bleeding at every meal, or when the tooth brush is used, causing foetid odors which are constantly taken into the lungs and causing the contamination of every particle of food that is taken into the stomach. Carious cavities become breeding places for pathogenic germs by affording lodgement for food which decomposes. Pus discharges from abscesses, and pyorrhea are frequently taken into the stomach for years successively, and when, at last, the person goes into a decline of health, the real cause of the trouble is overlooked.

Now, what is the relation of the physician to all of this? Simply this: The family physician should be the sentinel upon the watchtower of Zion. He should keep himself informed as to the physical condition of every member of the families over the care of whose health he is supposed to preside. In addition to making examinations of the hearts, lungs, livers, kidneys, etc., he should make careful, painstaking examinations of their mouths and insist upon them being kept in perfect condition. It is a cause for constant wonder on the part of the dentist who knows anything about the principles of surgery, to see how the physician who complies with all of the antiseptic precautions of modern surgery in the removal of every germ from his hands and field of operation in surgical cases, should be willing for his patient to take his nourishment and medicine through a mouth which, in many cases, is little better than a sewer, and advise expensive visits to watering places and other health resorts, when the same amount of money, and frequently less, invested in placing the oral cavity in a healthy condition, so that it can properly perform its natural functions, would give a much longer lease on life, and incidentally make them able for a much longer period to pay doctor bills.

Of course, when these conditions come under the observation of the dentist, he calls attention to them, but many dentists do not have the medical knowledge to appreciate the far-reaching effects of these things, and the dentist frequently does not see them until they are forced to him by pain, and in a condition beyond the reach of prophylactic treatment; besides, his advice is supposed to be selfish, while the word of the family physician carries the weight of authority with it.

The physician should be educated to know the significance of all of the conditions which exist in the oral cavity, and should be able to perform many of the operations, in case of necessity, which are relegated to the dental specialist, who, from the very nature of affairs, can not see the patient when confined at home; and this is especially true of the country practician.

That this is not done, is due to the faulty manner in which physical diagnosis is taught in our medical colleges, in which little attention is paid to conditions existing in the mouth—many of them not having chairs of dental and oral surgery—and in those which have, attention being called to only the grosser lesions, and numbers of conditions which are daily sapping the strength of the people, being entirely ignored.

The writer has received instruction in five different medical colleges, and in only one has any attention been given to the dental organs, and that principally directed to the extraction of teeth and the relief of toothache.

That, under the circumstances, the physician should have overlooked the gravity of neglected diseased conditions of the oral cavity and their far-reaching effects upon the general system, is not a subject for wonder.

